# Differential Expression and PAH Degradation: What *Burkholderia vietnamiensi*s G4 Can Tell Us?

**DOI:** 10.1155/2020/8831331

**Published:** 2020-08-27

**Authors:** Guilherme Pinto Cauduro, Ana Lusia Leal, Tiago Falcón Lopes, Marcela Marmitt, Victor Hugo Valiati

**Affiliations:** ^1^Universidade do Vale do Rio dos Sinos, Biology Graduate Program, São Leopoldo, RS, Brazil; ^2^Companhia Riograndense de Saneamento, Biology Laboratory, Triunfo, RS, Brazil; ^3^Centro de Terapia Gênica, Centro de Pesquisa Experimental, Hospital de Clínicas, Porto Alegre, RS, Brazil

## Abstract

Petroleum is the major energy matrix in the world whose refining generates chemical byproducts that may damage the environment. Among such waste, polycyclic aromatic hydrocarbons (PAH) are considered persistent pollutants. Sixteen of these are considered priority for remediation, and among them is benzo(a)pyrene. Amid remediation techniques, bioremediation stands out. The genus *Burkholderia* is amongst the microorganisms known for being capable of degrading persistent compounds; its strains are used as models to study such ability. High-throughput sequencing allows researchers to reach a wider knowledge about biodegradation by bacteria. Using transcripts and mRNA analysis, the genomic regions involved in this aptitude can be detected. To unravel these processes, we used the model *B. vietnamiensis* strain G4 in two experimental groups: one was exposed to benzo(a)pyrene and the other one (control) was not. Six transcriptomes were generated from each group aiming to compare gene expression and infer which genes are involved in degradation pathways. One hundred fifty-six genes were differentially expressed in the benzo(a)pyrene exposed group, from which 33% are involved in catalytic activity. Among these, the most significant genomic regions were phenylacetic acid degradation protein paaN, involved in the degradation of organic compounds to obtain energy; oxidoreductase FAD-binding subunit, related to the regulation of electrons within groups of dioxygenase enzymes with potential to cleave benzene rings; and dehydrogenase, described as accountable for phenol degradation. These data provide the basis for understanding the bioremediation of benzo(a)pyrene and the possible applications of this strain in polluted environments.

## 1. Introduction

Polycyclic aromatic hydrocarbons (PAHs) have been increasingly released into the environment by incomplete combustion of organic materials. This group of contaminants has harmful biological effects such as carcinogenicity, mutagenicity, and genotoxicity [1, 2] and it stands out as some of the most persistent pollutants in nature. They are classified as Persistent Organic Pollutants (POPs) of which 16 are recognized as priority for remediation by the US Environmental Protection Agency (USEPA) for having high toxicity levels to human health [1], including the benzo(a)pyrene used in this study, characterized by their stability and difficult degradation due to their chemical structure (aromatic rings) and their hydrophobicity [3–5]. Despite these properties, as they have genes that are expressed differently in the presence of these compounds [6, 7], a variety of bacteria can use them as a source of carbon and energy through their own metabolic pathways, playing a role in recycling the carbon of aromatic rings [8], degrading such pollutants [1].

Studies have shown that the enzymes, proteins, and metabolic pathways responsible for the biodegradation of PAHs can be produced in larger quantities, thus increasing the energy gain of these bacteria and their survival rate, making them adaptable to these environments [9–12]. De Gannes [13], in his study on genetic adaptations, presents three ways in which bacteria can survive in an environment with PAHs, such as carbon assimilation mediating enzymes, regulatory pathways for the expression of these enzymes, and modifications in cell structures for the absorption of compounds. Differential expression analysis and bioremediation experiments using these microorganisms allow us to detail this adaptation processes [14–17].

The *Burkholderia cepacia* complex (BCC) has been used as a model for the study of biodegradation [18]. This complex has at least nine species: *B. cepacia*, *B. multivorans*, *B. cenocepacia*, *B. vietnamiensis*, *B. stabilis*, *B. ambifaria*, *B. dolosa*, *B. anthina*, and *B. pyrrocinia*, which are genetically distinct and phenotypically similar [19]. They present great phenotypic plasticity and ecological versatility due to the size of their genome (4–9 Mb) [19]. *Burkholderia vietnamiensis* G4 [19] has been associated with organic pollutants since 1986, when its ability to metabolize trichloroethylene was recognized [20]. It has biotechnological potential and is used as a model of biodegradation due to its ability to degrade benzene, o-cresol, p-cresol, phenol, toluene, chloroform, benzo(a)pyrene, and naphthalene [21–24].

The *B. vietnamiensis* G4 capacity to survive harmful compounds in contaminated areas allowed its use in bioremediation experiments, combined with high-performance sequencing techniques and transcription analysis, which enabled the identification of genes differently expressed in its genome [23, 25–27] and its functions in the biodegradation process [28–31]. The use of new high-performance sequencing tools and transcriptome analysis is important to help us understand microorganism responses under different stress conditions [32]. In this context, the present study performed transcriptomic analyses in populations of *Burkholderia vietnamiensis* G4 exposed to benzo(a)pyrene with the aim to reveal the genes related to the degradation of this compound.

## 2. Materials and Methods

### 2.1. Bacterial Growth and RNA Extraction

The model selected for the differential expression tests was *Burkholderia vietnamiensis* strain G4 (Gram-negative, aerobic). This bacterium was chosen because it has already been described as presenting biodegradation capacity of several organic compounds. Its genome has been fully sequenced, which facilitates and enables a more detailed exploration of the data obtained with the differential gene expression experiment.

As a model for PAH, benzo(a)pyrene was chosen because it is one of the priority pollutants for remediation; it has five benzene rings making it exceedingly difficult to degrade, and it has high carcinogenic potential.

Initial bacterial growth was performed in liquid LB culture medium for 24 hours. Subsequently, the number of bacteria of 10^8^ cells/mL was standardized, using a spectrophotometer and absorbance reading at 625 nm. From this culture, the experimental stage of exposure of the bacteria to benzo(a)pyrene was initiated. Six Erlenmeyer culture flasks with 150 mL of Minimum Mineral Medium enriched with 10 mg/L of glucose were used, on which 1 mL of bacterial culture was added. In three of these, 1 mg/L of benzo(a)pyrene was added—characterizing the experimental group exposed to PAH (Bap1, Bap2, and Bap3). In the other three cultures, there was no addition of benzo(a)pyrene, and these were considered an unexposed control group (Neg1, Neg2, and Neg3) for expression comparisons. Both cultures, experimental and control, were incubated for 45 minutes at 30ºC and shaken at 140 rpm. The time of exposition was set to allow the bacteria to enter the growth phase, but not reach the duplication phase, thus avoiding production of RNAs involved in cell duplication.

After 45 minutes, all cellular processes were interrupted with the ProtectCell® kit (QIAGEN, Germany), increasing the stability of the extracted materials. The extraction of total RNA was performed with the RNeasy Mini Kit® (QIAGEN, Germany), following the manufacturer's recommendations. For each culture, two pools of total RNA were extracted and enriched with the Ribo-Zero rRNA Removal Kit-Bacteria® (Illumina®), producing twelve mRNA libraries, six from the experimental group and six from the control group.

### 2.2. RNA Sequence (RNA-Seq)

The sequencing of the twelve RNA libraries was performed on the single-end Illumina® (MiSeq) platform. The libraries were built using specific tags, following the manufacturer's instructions (TruSeq TMRNA® (Illumina®)).

### 2.3. Library Analysis

For the library analysis, samples originated from the same Erlenmeyer flask (two sequences) formed a single sample unit, with three experimental samples exposed to benzo(a)pyrene and three nonexposed control samples.

Sequences were filtered considering the Phred quality ≥ 30, which represents a 99% hit in the recognition of the bases. The Phred quality system is calculated by the probability of a base having been erroneously sequenced, given by the formula *q* = −10 × log(*p*), where *p* represents such probability. If an error is expected every one thousand sequenced bases, the Phred 30 value represents the expected error estimated at sequencing [33]. The quality verification of the filtered reads was performed with FastQC software version 0.11.3 [34, 35]. The reads were mapped by alignment with TopHat program version 2.0.9 (http://tophat.cbcb.umd.edu), which uses the files generated in the sequencing, aligning the reads in the indexer assembled with the genome (GenBank assembly accession: GCA_000016205.1) of the bacteria and its annotation file through Bowtie software version 1.1.1 (http://bowtie-bio.sourceforge.net/index.shtml).

### 2.4. Differential Expression Analysis

The quantification of the genes involved in the degradation capacity of benzo(a)pyrene, after alignment with TopHat, was performed with the software Cufflinks v. 2.1.1 (http://cufflinks.cbcb.umd.edu) [34]. The Cuffmerge extension was used to integrate the reads to TopHat mapping. Then, through the Cuffdiff tool, the levels of gene expression were verified and the statistical significance for the experimental and control groups was tested. Using the R (v. 3.3.2) package, CummeRbund version 0.1.3 (http://compbio.mit.edu/cummeRbund/), the analysis regarding the levels of expression was accessed [36]. To measure the significance of the differential expression, the corrected *p* values (*q*-value) were used, considering the False Discovery Rate (FDR) < 0.05. All plots and relationship evaluations of the genes significantly differentially expressed were generated with the gplots package.

Clusters analysis was performed using the pvclust package version 2.0.0 [37], based on the correlation distance associated with a complete connection method with 1000 replications of the bootstrap type. Correlation methods are highly recommended for gene expression data [38]. To verify the significance measurements, the corrected *p* values and the bootstrap were used as significance measurements, considering as significant the clusters with bp > 95% [37].

## 3. Results and Discussion

The sequencing resulted in a total of 19,433,337 sequences of the experimental group (Bap1—6,432,276; Bap2—8,113,328; and Bap3—4,887,733) and 20,802,491 sequences of the control group (Neg1—6,756,396; Neg2—5,724,941; and Neg3—8,321,154). Both groups were grown in the presence of glucose, whereas benzo(a)pyrene was only present in the experimental group. Even though several works choose to use the compound of interest as their sole source of carbon, we choose to use glucose as an additional carbon source for both optimizing growth and approximate environment conditions. Previous unpublished experiments from our group already demonstrate the ability of *Burkholderia vietnamiensis* G4 to grow using benzo(a)pyrene as the sole source of carbon. However, the intended application of this bacterial ability is to use it for bioremediation, a condition in which we would hardly find a single carbon source.

**Figure 1:**
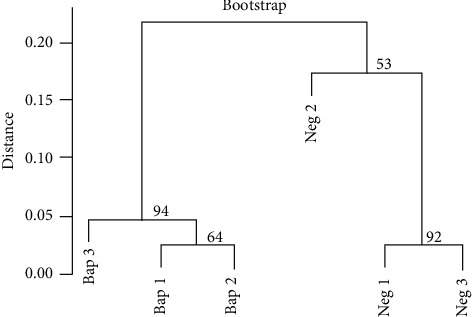
Gene-based correlation dendrogram between the experimental (Bap) and the control groups (Neg). The number in the branches indicates bootstrap support.

Evaluating the generated sequences, as well as the expressed genes, cluster analysis by correlation demonstrated well-supported clusters (bootstrap probability >90), forming clades in both the experimental and control groups (Figure 1).

**Figure 2:**
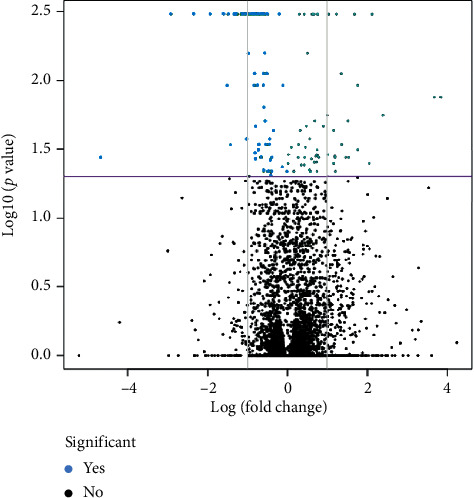
Volcano plot highlighting the genes differentially expressed between the group exposed to benzo(a)pyrene and the control group. A total of 156 differentially expressed genes (*p* < 0.05) were detected. In blue, we show the genes that presented statistically significant difference comparing the expression between both groups.

The sequences were mapped in the reference indexer built from the complete genome and its reference file for the genomic regions, finding 7,840 genes from the total described for the bacterium of 7,861 representing 99% coverage in the mapping of the generated reads. Of the 7,840 genes mapped, we found significant values of differential expression for 156 regions when comparing the experimental group to the control group (Supplementary Table 1). In Volcano (Figure 2), the significant genes for differential gene expression had a higher Fragments Per Kilobase Million (FPKM) value in the group of bacteria exposed to benzo(a)pyrene, totaling 88 higher expressed genes (Table 1), indicating greater expression of these genes in the experimental group. Of the genes expressed, we found 34 hypothetical proteins, and there were 13 genes expressed in clusters—for evaluation, they were considered as a single route of degradation.

**Figure 3:**
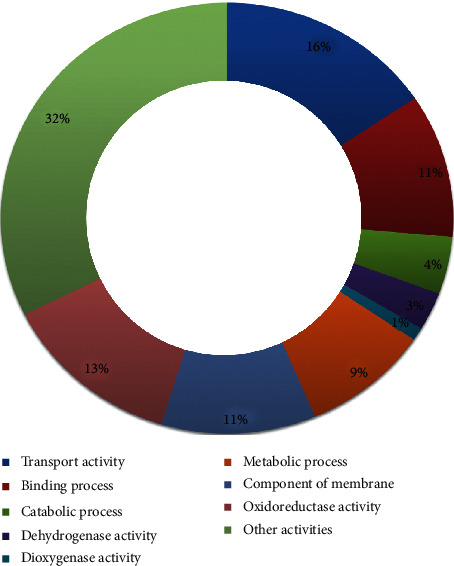
Distribution of genes differentially expressed by functional groups (GO Term).

The differentially expressed genes observed in this study in the *B. vietnamiensis* strain G4 showed involvement in several functions of this microorganism, which is an indicative of high metabolic activity of this bacterium when in contact with benzo(a)pyrene. From the genetic functionalities found in the differently expressed genomic regions, several functional groups were found (hydrolases, oxidoreductase, lyases, and transferases). This diversity of expressed regions demonstrates high metabolic activity in the group exposed to benzo(a)pyrene. Among the candidate genes found, it was observed that they correspond to a variety of functional groups. Nevertheless, groups of genes characterized by their involvement in catalytic activities represented most differentially expressed genes (Figure 3). These enzymes are associated with diverse biochemical and metabolic pathways, as an integral part of some of the most important life processes of these organisms [39]. The success of degradation of PAHs by these enzymes is directly linked to the ionization potential of each molecule, which represents the energy required for the enzymes to be able to act by removing electrons from the aromatic rings, consequently reducing its structural stability leading to the formation of simpler compounds and/or the breakdown of these PAHs [40–42].

Eighty-eight genes were found to have significant differential expression in the experimental group. They were associated with more than one cellular route for the degradation of PAHs. We also found regulatory regions that do not allow the byproducts resulting from this breakdown, such as CO_2_, H_2_O, ammonia, nitrogen, and potential charges of free electrons, which alter the intracellular pH and may lead to toxicity, to accumulate [43–45]. These regulatory regions provide the biological conversion of the toxic substrate generated by PAHs into some intermediate metabolites, such as acetyl-CoA, succinyl-CoA, and pyruvate, eliminating their cellular toxicity while maintaining their physiological functioning [46].

In order to reach successful degradation of PAHs, several cellular mechanisms must be active; thus, the expression of many functional groups, several proteins and enzymes, is expected. Among these, there are some membrane proteins that regulate the entry and exit of solutes and that were significantly highly expressed. Amid them, the *major facilitator transporter* (Bcep1808_2766) belongs to one of the largest super families of membrane transport proteins, also present in groups of bacteria, archaea, and eukaryotes. This family is important as an entry channel for micro- and macromolecules in the intracellular environment, as well as the release of dispensable substances [47], and may be active in the transport of benzo(a)pyrene molecules from the extracellular medium to the intracellular medium. The proteins *amino acid ABC transporter* (Bcep1808_0095, Bcep1808_3624, Bcep1808_5570, and Bcep1808_5571), *oligopeptide/dipeptide ABC transport ATPase* (Bcep1808_3703, Bcep1808_3703), *glycine betaine/L-proline ABC transport ATPase* (Bcep1808_3472), and *ABC transporter* (Bcep1808_5572) were all found to have a similar function. These proteins carry solutes and toxins through the membrane, regardless of the concentration gradient. Such movement occurs via ATP hydrolysis and it is fundamental to maintain cellular homeostasis [48]. Shuona et al. [49] found a positive regulation between transmembrane transport and the degradation of benzo(a)pyrene with the expression of certain proteins such as ABC transporter. The authors also highlight the relationship between the high hydrophobicity of the cell surface and the Gram-negative bacteria, such as *B. vietnamiensis* G4, which facilitates contact with benzo(a)pyrene.

Membrane activity is important for understanding the functioning and use of bacterial compounds. In this regard, a greater expression of the protein *lysine exporter protein LysE/YggA* (Bcep1808_3754) was observed among the bacterial strains of the group exposed to benzo(a)pyrene. This family of proteins, according to Tsu and Saier [50], presents the capacity of transmembrane transportation of specific metals, such as nickel, iron, and magnesium. The authors also highlight the importance of cell homeostasis, regulating and protecting the cell from the accumulation of heavy metals, which may be an important factor for bacteria that live in environments affected by persistent organic and inorganic compounds.

In the groups of transport and membrane proteins with differential expression, it is worth noting the *binding-protein-dependent transport system* (Bcep1808_3704, Bcep1808_3705, and Bcep1808_3471), described by Higgins et al. [51], the *extracellular solute-binding protein* (Bcep1808_0449, Bcep1808_4396, Bcep1808_3706, and Bcep1808_5569), and the *oxidoreductase FAD-binding subunit* (Bcep1808_3415). The latter plays an important role in electron chain flow regulation, linked to a larger metabolic system where the use of enzymes such as dioxygenases stands out [52, 53]. These enzymes form a super family of catalytic activity, and they are linked to the breakdown and transformation of organic compounds; the “Heterocyclic Ring Cleavage” [54] shows the importance of dioxygenases for the cleavage of aromatic rings and formation of carbon-carbon bonds, which are essential for the degradation of benzo(a)pyrene. Wang et al. [55] also show the importance of dioxygenases in breaking the aromatic rings, as in their experiment, these proteins were highly expressed only in the group exposed to pyrene. Many bacterial strains use these enzymes to obtain energy and carbon for their biological processes [56]. They were first isolated and characterized in *Pseudomonas putida* [57], a phylogenetically species closely related species to *B. vietnamiensis*, and the increased expression of this oxidoreductase is another indication of the use of benzo(a)pyrene as an energy source by *B. vietnamiensis*. Within the group of oxidoreductases, increased gene expression was detected for the proteins *glutamate synthase* (Bcep1808_0384, Bcep1808_0385), *methylitaconate delta2-delta3-isomerase* (Bcep1808_3677), *aldo/keto reductase* (Bcep1808_3756), and *FAD-dependent oxidoreductase* (Bcep1808_0754).

Regions also linked to dioxygenases, such as *4-hydroxy-phenylpyruvate-dioxygenase* (Bcep1808_0303) and *indolepyruvate-ferredoxin-oxidoreductase* (Bcep1808_0301), were already described in studies of bacteria surviving in stressful and contaminated environments [58, 59], expecting greater expression in the experimental group exposed to benzo(a)pyrene.

Among the overexpressed genes observed in the presence of benzo(a)pyrene, gene products involved in important catalytic pathways of PAH degradation were obtained; some were already found in the study of Ma et al. [60]. These genes belong to the dehydrogenase groups: *Acyl-CoA dehydrogenase* (Bcep1808_3337), *aldehyde dehydrogenase* (Bcep1808_6786), *methylmalonate-semialdehyde dehydrogenase* (Bcep1808_3335), and *trifunctional transcriptional regulator/proline dehydrogenase/pyrroline-5-carboxylate dehydrogenase* (Bcep1808_0122). Proline is directly related to the presence of a stressing agent, in the case of benzo(a)pyrene, and its high expression contributes to the resistance of the microorganism in the medium [61]. In a study conducted by O'Sullivan et al. [18] with mutants for phenol degradation, genes such as *aldehyde dehydrogenase* were interrupted, which showed an effect on phenol degradation. Changes in these enzymes indicated that they have an important support for this function. The fact that this route is also more expressed in the groups in contact with benzo(a)pyrene reinforces the hypothesis of using these enzymes as necessary for the degradation of organic aromatic compounds in these bacterial groups [62, 63].

The *phenylacetic acid degradation* protein is at the center of a route in the catabolism of aromatic compounds, converting the breakdown of these rings into energy directly in the Krebs cycle with the help of enzymes such as *phenylacetate-CoA ligase* [64–66]. In this study, we found four differentially expressed genes linked to this region: *phenylacetate-CoA ligase* (Bcep1808_0516), *phenylacetate-CoA oxygenase/reductase subunit PaaK* (Bcep1808_0308), *phenylacetic acid degradation protein PaaD* (Bcep1808_0517), and *phenylacetic acid degradation protein paaN* (Bcep1808_052). Therefore, the high expression of that route also places it as a strong candidate for benzo(a)pyrene degradation capacity [67] of *B. vietnamiensis* to obtain energy.

An important genomic region responsible for signaling degradation is the *XRE family transcriptional regulator* (xenobiotic response element) (Bcep1808_5376). This element is frequent in the regulation and activation of many metabolic pathways in bacteria and is involved in fitness and survival to stressful environments [68–70]. It is also linked to the activation of several pathways with capacity to use toxic compounds [71] active in bacteria when in contact with these compounds, such as the one used in the experiment, activating a system called toxin-antitoxin, which has a cascading effect that can generate production of biofilm, membrane enzymes, proteins for protection, growth, and cell signaling [72]. Considering that this is an element of resistance and activation of metabolic pathways, its increased expression makes it a good candidate to regulate the degradation of PAHs.

Four hypothetical proteins found are considered by us to represent the new candidates detected to play an important role in the degradation of benzo(a)pyrene. Searching for analogies and super families, the functional domain Dodecin was found for one of the hypothetical proteins (Bcep1808_1880). This domain is linked to small membrane molecules of the flavoproteins group, active in oxireduction and disposal pathways of cellular material [73, 74]. They are linked to processes of oxireduction of pyruvate, fatty acids and in the electron transport in energy chains. The biotransformation of PAHs involves a series of enzymes that catalyze oxidation, reduction, and hydrolysis reactions and enzymes that catalyze conjugation reactions. Xu et al. [75] indicate that dioxygenase can be used as biomarkers to assess initial oxidation and attenuation of HPAs and in their work naphthalene dioxygenase is used for this purpose. In general, the degradation of benzo(a)pyrene produces active intermediate metabolites such as dihydrodiol and dihydrodiol-epoxide [76, 77]. Such transformation can be interpreted as a facilitator of excretions by making a given metabolite more hydrophilic than its precursor. The induction of metabolic pathways such as these by chemicals, in the specific case by benzo(a)pyrene, may be suggestive of the participation of this hypothetical protein in the degradation of this PAH.

In studies involving benzo(a)pyrene degradation [1, 78–87], a great diversity of pathways, proteins and enzymes, are described. Among these, two large groups, dioxygenases and dihydrodiol, are the most cited as important in the degradation of benzo(a)pyrene as well as pyrene in the study of Wang et al. [55]. In our study, we found indications for the expression of dioxygenases. Meanwhile, the dihydrodiol groups did not show greater expression in this experiment, and it is speculated that this may be because these molecules are also expressed in the control group, even though it is not in contact with benzo(a)pyrene. We considered their involvement in other metabolic pathways and/or they might need longer than 45 minutes of exposure to be signaled and produced as possibilities for the nonsignificant expression of the dihydrodiol group in *B. vietnamiensis* G4.

## 4. Conclusion

In this study, the *B. vietnamiensis* G4 bacterium was exposed to the PAH benzo(a)pyrene, and the genes differentially expressed when in contact with this compound were evaluated. A total of 156 differentially expressed genes were found, 88 of which showed higher expression in the experimental group. Such high number of genes expressed suggests the possibility of several pathways of degradation of this compound. Considering that PAHs are persistent and highly harmful pollutants to human health and the environment, benzo(a)pyrene as one of the most toxic, the optimization of bioremediation processes is of fundamental importance [1, 2]. The mitigation of effects of these compounds in nature is essential for the conservation of ecosystems that are increasingly affected by PAHs, due to a growing use of oil derivatives and consequent increase of the global demand for their refining and processing, generating higher quantities of these compounds disposed in nature. Experiments with exposure of bacteria with capacity for degradation of persistent compounds, such as benzo(a)pyrene, test bacterial strains against model residual elements, thus verifying the ability of these compounds to be used by these microorganisms. The differential expression tests are informative to understand this bacterial capacity to degrade/assimilate and use these compounds in their metabolic pathways.

The evidence found in this study reveals some candidate genes for the degradation capacity of benzo(a)pyrene, a compound considered of difficult degradation. Understanding these processes can be useful for future efforts of increased degradation through genetic engineering and controlled overexpression of target genes.

## Figures and Tables

**Table 1:** Genes significantly expressed in the experimental group exposed to benzo(a)pyrene.

UniProtKB accession	NCBI old locus Tag	Coordinates	Gene length	Gene name	Product description	Significant in BAP
A4JK62	Bcep1808_3680	427848..428741(-−)	894	prpB	2-Methylisocitrate lyase	Yes
A4JAP4	Bcep1808_0334	378768..379043(+)	276	rpsS	30S ribosomal protein S19	Yes
A4JJM7	Bcep1808_3492	203642..205642(−)	2001		3-Methylcrotonoyl-CoA carboxylase subunit alpha	Yes
A4JDT5	Bcep1808_1430	1537771..1538475(+)	705		3-Oxoacid CoA-transferase subunit A	Yes
A4JAL3	Bcep1808_0303	336630..337727(−)	1098	hppD	4-Hydroxyphenylpyruvate dioxygenase	Yes
A4JBB7	Bcep1808_0558	614619..614882(+)	264	rpmA	50S ribosomal protein L27	Yes
A4JQF7	Bcep1808_5572	122728..123519(+)	792		ABC transporter related	Yes
A4JA13	Bcep1808_0093	100965..101744(+)	780		ABC transporter-like protein	Yes
A4JA20	Bcep1808_0100	109204..110988(+)	1785		ABC transporter-like protein	Yes
A4JQ98	Bcep1808_5507	63088..64278(+)	1191		Acetyl-CoA acetyltransferase	Yes
A4JK60	Bcep1808_3678	423951..426545(−-)	2595		Aconitate hydratase	Yes
A4JJ73	Bcep1808_3337	29534..30667(−)	1134		Acyl-CoA dehydrogenase	Yes
A4JTS0	Bcep1808_6786	182448..183836(−)	1389		Aldehyde dehydrogenase	Yes
A4JKD6	Bcep1808_3756	501291..502235(+)	945		aldo/keto reductase	Yes
A4JJ72	Bcep1808_3336	27598..29310(-)	1713		AMP-dependent synthetase/ligase	Yes
A4JQ96	Bcep1808_5505	60596..62263(+)	1668		AMP-dependent synthetase/ligase	Yes
A4JCX9	Bcep1808_1121	1218744..1219214(+)	471		AsnC family transcriptional regulator	Yes
A4JB78	Bcep1808_0519	561462..562664(−)	1203		Beta-ketoadipyl CoA thiolase	Yes
A4JJK6	Bcep1808_3471	173048..173944(−)	897		Binding-protein-dependent transport system inner membrane protein	Yes
A4JK86	Bcep1808_3704	454654..455556(−)	903		Binding-protein-dependent transport system inner membrane protein	Yes
A4JK87	Bcep1808_3705	455570..456508(−)	939		Binding-protein-dependent transport system inner membrane protein	Yes

## Data Availability

The underlying data supporting the results of their study, in addition to the supplementary materials available in the submitted document, are available and can be requested by sending e-mails to valiati@unisinos.br and gcauduro@gmail.com.

## Supplementary Materials

Supplementary Materials Supplementary Table 1. The 156 genes that were found with significant values of differential expression when we compared the experimental group with the control group.
