# Supplementary material for: Differential Expression and PAH Degradation: What Burkholderia vietnamiensis G4 Can Tell Us?
Source: Int J Microbiol. 2020 Aug 27;2020:8831331. doi: 10.1155/2020/8831331 (PMC7474390; doi:10.1155/2020/8831331)
Supplement: Supplementary Materials — Supplementary Table 1. The 156 genes that were found with significant values of differential expression when we compared the experimental group with the control group. [file 8831331.f1.docx]

Supplementary table 1: The 156 genes that were found with significant values of differential expression when we compared the experimental group with the control group.

| **UniProtKB Accession** | **NCBI Old Locus Tag** | **Coordinates** | **Gene Length** | **Gene Name** | **Product Description** | **Significant in BAP** |
| --- | --- | --- | --- | --- | --- | --- |
| A4JA15 | Bcep1808_0095 | 102574..103773(+) | 1200 |  | branched chain amino acid ABC transporter periplasmic ligand-binding protein | Yes |
| A4JDT6 | Bcep1808_1431 | 1538477..1539118(+) | 642 |  | butyryl-CoA:acetate CoA transferase | Yes |
| A4JK06 | Bcep1808_3624 | 366686..367468(+) | 783 |  | cationic amino acid ABC transporter, periplasmic binding protein | Yes |
| A4JB44 | Bcep1808_0485 | 526970..527173(-) | 204 |  | cold-shock DNA-binding protein family protein | Yes |
| A4JD64 | Bcep1808_1206 | 1306289..1307719(+) | 1431 |  | cytosine/purines, uracil, thiamine, allantoin permease | Yes |
| A4JB77 | Bcep1808_0518 | 560625..561416(-) | 792 |  | enoyl-CoA hydratase | Yes |
| A4JA18 | Bcep1808_0098 | 106824..107981(+) | 1158 |  | extracellular ligand-binding receptor | Yes |
| A4JAF1 | Bcep1808_0238 | 266802..267944(+) | 1143 |  | extracellular ligand-binding receptor | Yes |
| A4JCU1 | Bcep1808_1083 | 1168585..1169733(-) | 1149 |  | extracellular ligand-binding receptor | Yes |
| A4JB08 | Bcep1808_0449 | 480091..480891(-) | 801 |  | extracellular solute-binding protein | Yes |
| A4JK88 | Bcep1808_3706 | 456520..458133(-) | 1614 |  | extracellular solute-binding protein | Yes |
| A4JM59 | Bcep1808_4396 | 1221013..1222059(+) | 1047 |  | extracellular solute-binding protein | Yes |
| A4JQF4 | Bcep1808_5569 | 120266..121126(+) | 861 |  | extracellular solute-binding protein | Yes |
| A4JBW3 | Bcep1808_0754 | 827159..828826(+) | 1668 |  | FAD-dependent oxidoreductase | Yes |
| A4JBH6 | Bcep1808_0617 | 675347..675775(-) | 429 |  | ferric uptake regulator family protein | Yes |
| A4JBW7 | Bcep1808_0758 | 831802..833106(+) | 1305 |  | fumarylacetoacetate hydrolase | Yes |
| A4JPM2 | Bcep1808_5277 | 2203737..2205488(-) | 1752 |  | gamma-glutamyltransferase 1 | Yes |
| A4JE07 | Bcep1808_1503 | 1620050..1620592(+) | 543 |  | GCN5-like N-acetyltransferase | Yes |
| A4JAU5 | Bcep1808_0385 | 423628..425094(+) | 1467 | gltD | glutamate synthase subunit beta | Yes |
| A4JJK7 | Bcep1808_3472 | 173937..175106(-) | 1170 |  | glycine betaine/L-proline ABC transporter ATPase | Yes |
| A4JL44 | Bcep1808_4016 | 800698..801897(+) | 1200 |  | GntR family transcriptional regulator | Yes |
| A4JA51 | Bcep1808_0131 | 146633..148768(+) | 2136 |  | heavy metal translocating P-type ATPase | Yes |

Supplementary table 1: Continuation

| **UniProtKB Accession** | **NCBI Old Locus Tag** | **Coordinates** | **Gene Length** | **Gene Name** | **Product Description** | **Significant in BAP** |
| --- | --- | --- | --- | --- | --- | --- |
| A4JIG3 | Bcep1808_3075 | 3408358..3408558(+) | 201 |  | heavy metal transport/detoxification protein | Yes |
| A4JT56 | Bcep1808_6564 | 1179907..1180218(+) | 312 |  | high potential iron-sulfur protein | Yes |
| A4JQF8 | Bcep1808_5573 | 123527..125020(+) | 1494 |  | histidine ammonia-lyase | Yes |
| A4JQF9 | Bcep1808_5574 | 125065..125820(+) | 756 |  | histidine utilization repressor | Yes |
| A4JAC0 | Bcep1808_0207 | 233365..233661(+) | 297 |  | histone family protein nucleoid-structuring protein H-NS | Yes |
| A4JAF4 | Bcep1808_0241 | 270584..270877(+) | 294 |  | histone family protein nucleoid-structuring protein H-NS | Yes |
| A4JBW6 | Bcep1808_0757 | 830471..831805(+) | 1335 |  | homogentisate 1,2-dioxygenase | Yes |
| A4JPD0 | Bcep1808_5183 | 2095599..2095895(-) | 297 |  | hypothetical protein | Yes |
| A4JPD4 | Bcep1808_5187 | 2099017..2099346(+) | 330 |  | hypothetical protein | Yes |
| A4JPH0 | Bcep1808_5224 | 2141436..2141663(-) | 228 |  | hypothetical protein | Yes |
| A4JQ77 | Bcep1808_5486 | 35854..36180(+) | 327 |  | hypothetical protein | Yes |
| A4JAL1 | Bcep1808_0301 | 332420..336010(-) | 3591 |  | indolepyruvate ferredoxin oxidoreductase | Yes |
| A4JA16 | Bcep1808_0096 | 103836..104777(+) | 942 |  | inner-membrane translocator | Yes |
| A4JA19 | Bcep1808_0099 | 108075..109199(+) | 1125 |  | inner-membrane translocator | Yes |
| A4JKD4 | Bcep1808_3754 | 500378..500998(+) | 621 |  | lysine exporter protein LysE/YggA | Yes |
| A4JD65 | Bcep1808_1207 | 1307793..1308749(-) | 957 |  | LysR family transcriptional regulator | Yes |
| A4JHK4 | Bcep1808_2766 | 3060636..3062294(+) | 1659 | deh4p | major facilitator transporter | Yes |
| A4JJF7 | Bcep1808_3422 | 119390..120361(-) | 972 |  | membrane dipeptidase | Yes |
| A4JK61 | Bcep1808_3679 | 426627..427799(-) | 1173 |  | methylcitrate synthase | Yes |
| A4JK59 | Bcep1808_3677 | 422734..423924(-) | 1191 |  | methylitaconate delta2-delta3-isomerase | Yes |
| A4JJ71 | Bcep1808_3335 | 25926..27455(-) | 1530 |  | methylmalonate-semialdehyde dehydrogenase | Yes |
| A4JPU2 | Bcep1808_5347 | 2289215..2290861(-) | 1647 |  | natural resistance-associated macrophage protein | Yes |

Supplementary table 1: Continuation

| **UniProtKB Accession** | **NCBI Old Locus Tag** | **Coordinates** | **Gene Length** | **Gene Name** | **Product Description** | **Significant in BAP** |
| --- | --- | --- | --- | --- | --- | --- |
| A4JK84 | Bcep1808_3702 | 452629..453663(-) | 1035 |  | oligopeptide/dipeptide ABC transporter ATPase | Yes |
| A4JK85 | Bcep1808_3703 | 453660..454652(-) | 993 |  | oligopeptide/dipeptide ABC transporter ATPase | Yes |
| A4JGU3 | Bcep1808_2498 | 2765072..2766157(+) | 1086 |  | outer membrane protein (porin)-like protein | Yes |
| A4JJF0 | Bcep1808_3415 | 110198..111346(-) | 1149 |  | oxidoreductase FAD-binding subunit | Yes |
| A4JB75 | Bcep1808_0516 | 558834..560132(-) | 1299 |  | phenylacetate-CoA ligase | Yes |
| A4JAL8 | Bcep1808_0308 | 341007..342095(-) | 1089 |  | phenylacetate-CoA oxygenase/reductase subunit PaaK | Yes |
| A4JB76 | Bcep1808_0517 | 560171..560623(-) | 453 |  | phenylacetic acid degradation protein PaaD | Yes |
| A4JB79 | Bcep1808_0520 | 562761..564467(-) | 1707 |  | phenylacetic acid degradation protein paaN | Yes |
| A4JB79 | Bcep1808_0520 | 562761..564467(-) | 1707 |  | phenylacetic acid degradation protein paaN | Yes |
| A4JNU9 | Bcep1808_4996 | 1884380..1886245(-) | 1866 |  | phosphoenolpyruvate carboxykinase | Yes |
| A4JQF5 | Bcep1808_5570 | 121219..121941(+) | 723 |  | polar amino acid ABC transporter, inner membrane subunit | Yes |
| A4JQF6 | Bcep1808_5571 | 121954..122700(+) | 747 |  | polar amino acid ABC transporter, inner membrane subunit | Yes |
| A4JB83 | Bcep1808_0524 | 575713..576882(-) | 1170 |  | Porin | Yes |
| A4JJF1 | Bcep1808_3416 | 111376..112617(-) | 1242 |  | Rieske (2Fe-2S) domain-containing protein | Yes |
| A4JQW3 | Bcep1808_5729 | 314616..316049(+) | 1434 |  | RNA polymerase factor sigma-54 | Yes |
| A4JJF8 | Bcep1808_3423 | 120404..121678(-) | 1275 |  | serine hydroxymethyltransferase | Yes |
| A4JJF9 | Bcep1808_3424 | 122128..123156(+) | 1029 |  | transcriptional regulator | Yes |
| A4JF33 | Bcep1808_1883 | 2055543..2055896(+) | 354 |  | transthyretin | Yes |
| A4JA42 | Bcep1808_0122 | 133571..137500(+) | 3930 | putA | trifunctional transcriptional regulator/proline dehydrogenase/pyrroline-5-carboxylate dehydrogenase | Yes |
| A4JPX0 | Bcep1808_5376 | 2321649..2322206(-) | 558 |  | XRE family transcriptional regulator | Yes |
| A4JI23 | Bcep1808_2935 | 3256508..3256798(+) | 291 |  | YCII-related | Yes |

Supplementary table 1: Continuation

| **UniProtKB Accession** | **NCBI Old Locus Tag** | **Coordinates** | **Gene Length** | **Gene Name** | **Product Description** | **Significant in BAP** |
| --- | --- | --- | --- | --- | --- | --- |
| A4JHR4 | Bcep1808_2826 | 3131160..3133595(-) | 2436 |  | 3-hydroxyacyl-CoA dehydrogenase | Yes |
| A4JPJ6 | Bcep1808_5250 | 2165230..2166564(+) | 1335 |  | ABC-type phosphate transport system periplasmic component-like protein | No |
| A4JPZ4 | Bcep1808_5403 | 2352469..2355555(-) | 3087 |  | acriflavin resistance protein | No |
| A4JHR5 | Bcep1808_2827 | 3133709..3135496(-) | 1788 |  | acyl-CoA dehydrogenase domain-containing protein | No |
| A4JIE0 | Bcep1808_3052 | 3385399..3385890(+) | 492 |  | AsnC family transcriptional regulator | No |
| A4JIE6 | Bcep1808_3058 | 3391787..3392824(+) | 1038 |  | bile acid:sodium symporter | No |
| A4JPI1 | Bcep1808_5235 | 2150728..2151138(-) | 411 |  | biopolymer transport protein ExbD/TolR | No |
| A4JSZ4 | Bcep1808_6500 | 1106199..1110647(-) | 4449 |  | cellulose synthase domain-containing protein | No |
| A4JPD2 | Bcep1808_5185 | 2097682..2097999(-) | 318 |  | chaperonin Cpn10 | No |
| A4JKP8 | Bcep1808_3870 | 635359..636336(-) | 978 |  | chromate resistance exported protein | No |
| A4JKP7 | Bcep1808_3869 | 634184..635362(-) | 1179 |  | chromate transporter | No |
| A4JJV5 | Bcep1808_3571 | 306476..307840(+) | 1365 |  | citrate carrier protein | No |
| A4JH40 | Bcep1808_2598 | 2874090..2874293(+) | 204 |  | cold-shock DNA-binding protein family protein | No |
| A4JIB8 | Bcep1808_3030 | 3356248..3356973(-) | 726 |  | CutC family protein | No |
| A4JK34 | Bcep1808_3652 | 392706..394127(+) | 1422 |  | cytochrome bd ubiquinol oxidase, subunit I | No |
| A4JIC5 | Bcep1808_3037 | 3369125..3370018(+) | 894 |  | dihydrodipicolinate synthase | No |
| A4JV87 | Bcep1808_7313 | 10411..12855(-) | 2445 |  | DNA topoisomerase | No |
| A4JPJ0 | Bcep1808_5244 | 2160869..2161435(-) | 567 |  | ECF subfamily RNA polymerase sigma-24 factor | No |
| A4JCW3 | Bcep1808_1105 | 1195007..1195801(+) | 795 |  | extracellular solute-binding protein | No |
| A4JFD8 | Bcep1808_1988 | 2173224..2174846(+) | 1623 |  | FAD dependent oxidoreductase | No |
| A4JD92 | Bcep1808_1235 | 1335200..1336684(-) | 1485 |  | general substrate transporter | No |
| A4JBJ3 | Bcep1808_0634 | 694341..695627(+) | 1287 |  | Glu/Leu/Phe/Val dehydrogenase, C terminal | No |
| A4JAU4 | Bcep1808_0384 | 418839..423542(+) | 4704 |  | glutamate synthase (NADH) large subunit | No |

Supplementary table 1: Continuation

| **UniProtKB Accession** | **NCBI Old Locus Tag** | **Coordinates** | **Gene Length** | **Gene Name** | **Product Description** | **Significant in BAP** |
| --- | --- | --- | --- | --- | --- | --- |
| A4JBH1 | Bcep1808_0612 | 670154..671164(+) | 1011 |  | glyceraldehyde-3-phosphate dehydrogenase | No |
| A4JI17 | Bcep1808_2929 | 3247042..3247776(-) | 735 |  | GntR family transcriptional regulator | No |
| A4JE03 | Bcep1808_1498 | 1614134..1614427(+) | 294 |  | hypothetical protein | No |
| A4JE04 | Bcep1808_1499 | 1614458..1615378(-) | 921 |  | hypothetical protein | No |
| A4JF30 | Bcep1808_1880 | 2052223..2052432(+) | 210 |  | hypothetical protein | No |
| A4JIB2 | Bcep1808_3024 | 3349409..3349846(+) | 438 |  | hypothetical protein | No |
| A4JID1 | Bcep1808_3043 | 3377640..3377933(+) | 294 |  | hypothetical protein | No |
| A4JID3 | Bcep1808_3045 | 3380146..3380769(-) | 624 |  | hypothetical protein | No |
| A4JID9 | Bcep1808_3051 | 3384384..3385286(-) | 903 |  | hypothetical protein | No |
| A4JJH1 | Bcep1808_3436 | 136135..137745(-) | 1611 |  | hypothetical protein | No |
| A4JJM6 | Bcep1808_3491 | 202236..203378(-) | 1143 |  | hypothetical protein | No |
| A4JJN6 | Bcep1808_3501 | 215469..217391(-) | 1923 |  | hypothetical protein | No |
| A4JPH1 | Bcep1808_5225 | 2141836..2142300(-) | 465 |  | hypothetical protein | No |
| A4JPH3 | Bcep1808_5227 | 2143991..2144335(-) | 345 |  | hypothetical protein | No |
| A4JPH4 | Bcep1808_5228 | 2144403..2145353(-) | 951 |  | hypothetical protein | No |
| A4JPH6 | Bcep1808_5230 | 2146338..2146778(-) | 441 |  | hypothetical protein | No |
| A4JPH7 | Bcep1808_5231 | 2146775..2147350(-) | 576 |  | hypothetical protein | No |
| A4JPJ1 | Bcep1808_5245 | 2161442..2162179(-) | 738 |  | hypothetical protein | No |
| A4JPS5 | Bcep1808_5330 | 2257812..2271305(+) | 13494 |  | hypothetical protein | No |
| A4JV81 | Bcep1808_7307 | 4251..6275(-) | 2025 |  | hypothetical protein | No |
| A4JCD0 |  | 1002489..1002836(-) | 348 |  | hypothetical protein | No |
| A4JDV7 |  | 1561455..1561694(-) | 240 |  | hypothetical protein | No |
| A4JAJ8 |  | 317163..317465(+) | 303 |  | hypothetical protein | No |
| A4JP75 |  | 2035606..2035716(-) | 111 |  | hypothetical protein | No |

Supplementary table 1: Continuation

| **UniProtKB Accession** | **NCBI Old Locus Tag** | **Coordinates** | **Gene Length** | **Gene Name** | **Product Description** | **Significant in BAP** |
| --- | --- | --- | --- | --- | --- | --- |
| A4JP83 |  | 2043205..2043483(-) | 279 |  | hypothetical protein | No |
| A4JPC0 |  | 2085041..2085304(-) | 264 |  | hypothetical protein | No |
| A4JPF5 |  | 2125793..2125936(-) | 144 |  | hypothetical protein | No |
| A4JPG3 |  | 2133952..2134269(-) | 318 |  | hypothetical protein | No |
| A4JPG3 |  | 2133952..2134269(-) | 318 |  | hypothetical protein | No |
| A4JKD5 |  | 501002..501319(+) | 318 |  | hypothetical protein | No |
| A4JTY8 |  | 253172..253810(-) | 639 |  | hypothetical protein | No |
| A4JFD7 | Bcep1808_1987 | 2172308..2173111(+) | 804 |  | inositol monophosphatase | No |
| A4JPA4 | Bcep1808_5156 | 2069445..2070068(-) | 624 |  | lysine exporter protein LysE/YggA | No |
| A4JI65 | Bcep1808_2977 | 3298499..3299542(+) | 1044 |  | LysR family transcriptional regulator | No |
| A4JIC6 | Bcep1808_3038 | 3370358..3371413(-) | 1056 |  | LysR family transcriptional regulator | No |
| A4JIC7 | Bcep1808_3039 | 3371520..3372932(+) | 1413 |  | major facilitator transporter | No |
| A4JI56 | Bcep1808_2968 | 3285081..3286781(+) | 1701 |  | Mg chelatase subunit ChlI | No |
| A4JPA0 | Bcep1808_5152 | 2064839..2065417(-) | 579 |  | putative transmembrane protein | No |
| A4JNS6 | Bcep1808_4973 | 1856320..1856925(+) | 606 |  | putative zinc-containing dehydrogenase | No |
| A4JPZ5 | Bcep1808_5404 | 2355552..2356649(-) | 1098 |  | RND family efflux transporter MFP subunit | No |
| A4JIF4 | Bcep1808_3066 | 3400443..3401186(+) | 744 |  | short-chain dehydrogenase/reductase SDR | No |
| A4JCW2 | Bcep1808_1104 | 1193685..1194770(+) | 1086 |  | succinylglutamate desuccinylase | No |
| A4JM53 | Bcep1808_4390 | 1212357..1214486(+) | 2130 |  | TonB-dependent copper receptor | No |
| A4JJN5 | Bcep1808_3500 | 213245..215440(+) | 2196 |  | TonB-dependent siderophore receptor | No |
| A4JPI0 | Bcep1808_5234 | 2150025..2150723(-) | 699 |  | TonB-like protein | No |
| A4JBB1 | Bcep1808_0552 | 606733..607947(-) | 1215 |  | type II secretion system protein | No |
| A4JBB2 | Bcep1808_0553 | 607944..609188(-) | 1245 |  | type II secretion system protein E | No |
